# Case of Malformation

**Published:** 1877-01-20

**Authors:** 


### CASE OF MALFORMATION.

Dr. W. G. Hunt, of Kentucky, writes us that he had a singular case of imperfect development of the genitals in a fœtus:

"Nature, in one of her freaks, presented me with a very peculiar phenomenon in the way of monstrosity, by failing in her anatomical construction of the female genitals.

The child being born, my attention was called by the nurse, to make the necessary discrimination, usually called for by the parents, as to whether a male or female, as the characteristic developments were insufficient for a satisfactory diagnosis. "Why, Doc., is it a morphadite?" (hermaphrodite) inquired the old lady. I, of course, responded in the negative, as I think there is no such thing, unless the words hermaphrodite and monstrosity are synonymous. The labia majora presented the appearance and felt much like the male scrotum, there being perfect occlusion of the vulvæ, with a raphe separating the labia, similar to that of the male scrotum, extending to the meatus urinarus, presenting no marks of distinction of sex, except the clitoris and meatus urinarus. I expected to have operated in this case, but lost sight of the unfortunate child by its removal from my neighborhood. It ultimately died of malarial fever."

